# The research trends and hotspots of claudins in the field of cancer

**DOI:** 10.1080/19336918.2025.2520641

**Published:** 2025-06-20

**Authors:** Weixin Jing, Linna Long, Xiaorui Zhang, Xia Li, He Huang

**Affiliations:** a Department of Biology, School of Basic Medical Sciences, Xinjiang Medical University, Urumqi, China; b Xinjiang Key Laboratory of Molecular Biology for Endemic Diseases, Xinjiang Medical University, Urumqi, China; c Department of Histology & Embryology, Xiangya School of Medicine, Central South University, Changsha, China; d Department of Gynecology, Affiliated Tumor Hospital of Xinjiang Medical University, Urumqi, China

**Keywords:** Claudin, cancer, scientometric analysis, CiteSpace, Vosviewer

## Abstract

Claudins (CLDNs), as the key components of tight junctions, have been implicated as key factors in carcinogenesis and metastasis. A total of 1720 publications on CLDNs in the field of cancer were published from January 2005 to December 2022. The United States dominates the research on CLDNs in cancer, followed by China. China Medical University is the most productive, and Johns Hopkins University has the most citations. Morin PJ is credited with initiating research on CLDNs in cancer. CLDN18, the intestinal barrier, and the intestinal microbiota are the focus and hotspots in this field. The mechanism of CLDN-mediated metastasis still needs further investigation, and the development of CLDN-targeted therapies also needs to be explored in the future.

## Introduction

Cancer is known to have a high mortality rate and is difficult to cure. Ninety percent of human cancer deaths are caused by the metastases of tumor cells [1], which is a dynamic and multifaceted process during which the primary tumor sustains proliferative signaling, evades growth suppressors and the immune system, activates invasion, induces/accesses the vasculature, extravasates, and colonizes in a distant organ [2]. For successful metastasis, epithelial cells must lose their apical-basal polarity and cell – cell junctions, especially tight junctions (TJs), to activate invasion and access vasculature capabilities [3]. The biological process of epithelial cells transforming into mesenchymal phenotype cells is termed the epithelial – mesenchymal transition (EMT) [4]. TJs play an important role in activating the invasion and metastasis of cancer because the loss of intercellular junctions is a prerequisite [5].

Claudins (CLDNs), as the key components of TJs, form homo- and heteromeric interactions between adjacent cells, have paracellular permselective barrier and defense functions, and participate in cell signal transduction regulating cell growth, survival, proliferation, differentiation, and polarity [6,7]. CLDNs are a family of transmembrane proteins composed of four transmembrane domains, two extracellular loops, one intracellular loop and the cytoplasmic *N*- and C-termini, with molecular masses of 21–34 kDa [8,9]. Since CLDN1 and CLDN2 were first discovered in 1998 [10], there are thought to be 26 human CLDNs [11]. These CLDNs have high sequence homology and can be classified into two categories: classical CLDNs (CLDN 1–10, 14, 15, 17, and 19) and nonclassical CLDNs (CLDN 11–12, 16, 18, 20, and 22–25) [12].

The transcriptional expression of *CLDNs* has tissue specificity, varies considerably in normal and cancer tissues, and has been implicated as a key factor in carcinogenesis and metastasis [5,13]. There was a study combining serial analysis of gene expression (SAGE) databases and real-time RT-PCR technology to investigate the transcriptional expression of *CLDNs* in normal and tumor tissues [14]. The results showed that *CLDN1–7*, *CLDN11–12*, and *CLDN15* were expressed in multiple tissues. For example, *CLDN5* was expressed at high levels in vascular endothelial cells, suggesting a target for antiangiogenic therapy [14]. *CLDN6* was found to be expressed only in embryonic stem cells and might be related to early embryonic development [14]. Contrary to the low expression of most *CLDNs* in tumors, *CLDN3*, *CLDN4*, and *CLDN7* were highly expressed in several tumors, such as lung, pancreas, bladder, stomach, colon, breast, and prostate tumors [14].

Although the function and expression of CLDNs vary between normal and tumor tissues, this does not prevent some CLDNs from being used as diagnostic biomarkers and therapeutic targets due to their significant clinical relevance. The expression of CLDNs, such as CLDN1 in colon cancer [15], CLDN18 in gastric cancer [16], and CLDN10 in hepatocellular carcinoma [17] and metastatic high-grade serous carcinoma [18], has been shown to have prognostic value. Thus, CLDNs play an important role in tumorigenesis and development. To the best of our knowledge, no study has summarized the articles about research on CLDNs in cancer with scientometric research methods. Scientometric analysis can help us understand the research context, current research trends and hotspots and can help researchers determine the most influential publications, journals, and scholars [19]. In this study, the literature related to CLDNs in the field of cancer was searched, and bibliometric analysis was used to summarize and analyze research frontiers, hotspots, and trends.

## Methods

### Data source and collection

**Figure 1.**
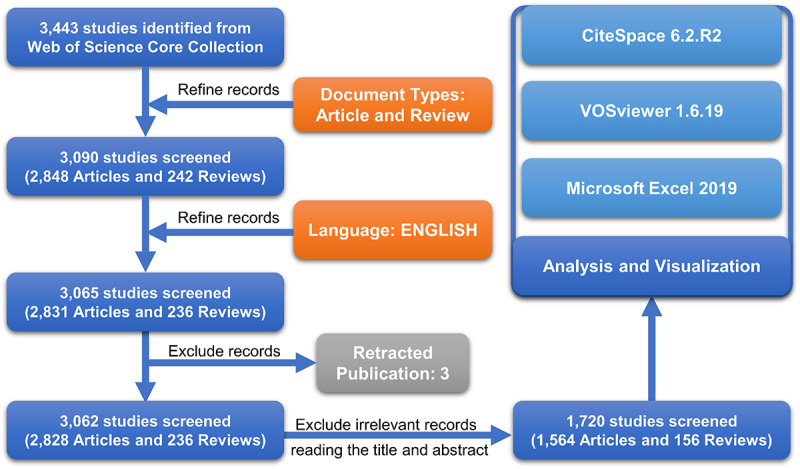
Publication screening and analysis flowchart.

Comprehensive literature research on CLDNs in the field of cancer was performed on the Science Citation Index Expanded (SCI-Expanded). The search strategies were as follows: TS = (‘cancer’ OR ‘tumour’ OR ‘neoplasm’ OR ‘carcinoma’ OR ‘adenocarcinoma’ OR ‘sarcoma’ OR ‘melanoma’ OR ‘oncology’) AND TS = (‘claudins’ OR ‘CLDNs’ OR ‘claudin’ OR ‘CLDN’) with the publication date from 1 January 2005, to 31 December 2022. Then, the document types were limited to Article or Review Article, and Retracted Publications were excluded. The languages were limited to English. Finally, 1720 publications were included after excluding irrelevant literature and were exported and stored in a plain text file (including full records and cited references) for scientometric analysis and visualization on 10 April 2023 (Figure 1).

### Scientometric analysis

Both CiteSpace and VOSviewer are often used for data analysis and visualization tools in scientometrics [20,21]. CiteSpace (6.2. R2) [22] was employed to obtain collaborative networks (countries/regions, institutions, and authors), co-cited networks (co-cited authors, co-cited journals, and co-cited references), co-occurrence networks of authors’ keywords, timeline visualization (co-cited authors, co-cited references, and authors’ keywords) and burst analysis (countries/regions, co-cited authors, co-cited references, and authors’ keywords). The radius of a node is made of tree rings and reflects the numbers of publications or citations, and the color indicates the average published year, cited year or cluster type. The different network analyses used by CiteSpace involve many different metrics, such as betweenness centrality, modularity, silhouette score, citation burstness, and a compound metric sigma [23]. Betweenness centrality calculates a node centrality in a network based on shortest paths [24]. High betweenness centrality indicates betweenness centrality of at least 0.1 and is represented by the thickness of the purple trim [19]. Modularity, the cluster module value (Q-value), measures the strength of the division of a network into clusters. Generally, Q > 0.3 indicates a significant cluster structure [25]. The silhouette score (S-value) calculates the goodness of a cluster. For S-values exceeding 0.5 or 0.7, the cluster is reasonable or highly credible, respectively [26]. Sigma (∑) is a compound metric with betweenness centrality and burstness indicating influential potential [22].

VOSviewer (1.6.19) [27] was used to extract collaborative networks of countries/regions and for density visualization of source journals. For collaborative analysis, at least five relevant publications were published per country/region. The minimum number of publications per journal was set at 10 in the density visualization of source journals. The size of a node reflects the number of publications or citations, and the color indicates the average published year, cited year or cluster type. An online bibliometric website (https://bibliometric.com) was employed to display a collaborative network of countries/regions. The size of the different color blocks indicates the number of publications published by the corresponding countries/regions. The journal impact factors (JIFs) were obtained from the 2021 Journal Citation Reports (JCR). The annual publications and citations and other basic data were analyzed and plotted using Microsoft Excel 2019.

## Results

**Figure 2.**
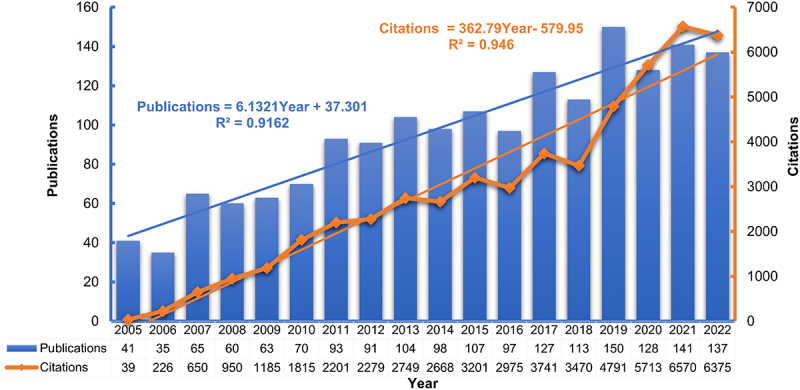
Annual publications and citations of global research on claudins in the field of cancer.

A total of 1720 publications on CLDNs in the field of cancer were published from January 2005 to December 2022, including 1564 (90.93%) articles and 156 reviews (9.07%). The relevant publications had an H-Index of 97, with 52,965 citations and 39,952 citations without self-citations (Figure 2). The number of publications increased from 41 publications in 2005 to 137 publications in 2022, with an average annualized growth rate of approximately 15%. Over time, the citations of these studies increased continuously. There were only 39 citations in 2005, increasing to 6375 citations in 2022, with an average annualized growth rate of over 1,000%.

### Countries/regions, affiliations, and authors

**Figure 3.**
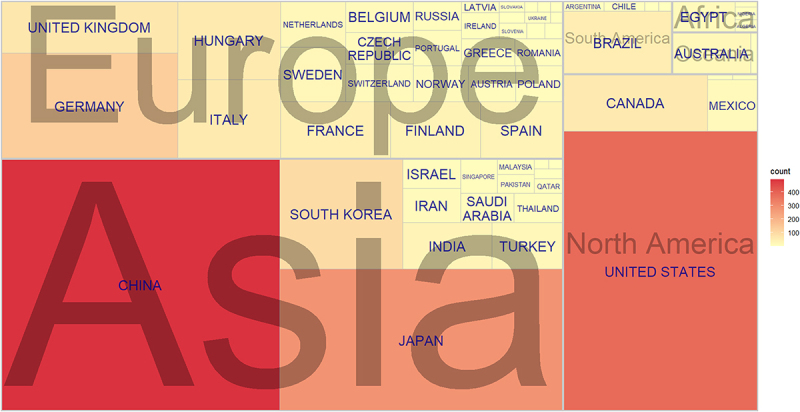
The countries/regions contributing to claudins in the field of cancer.

**Figure 4.**
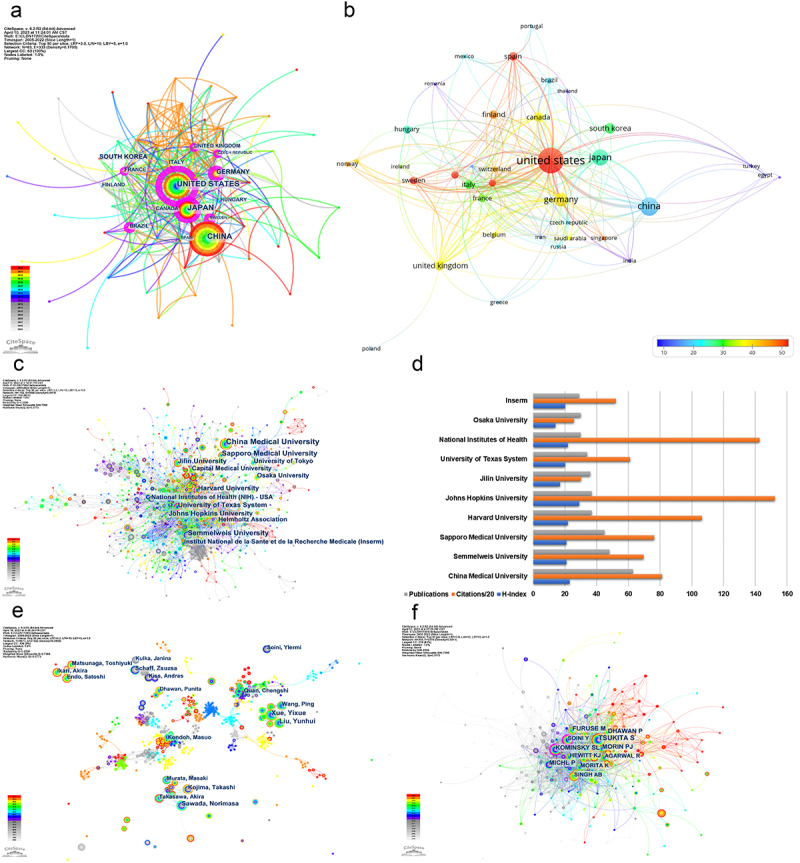
Collaborative networks of countries/regions, institutions, authors, and co-cited authors. (a) Overlay visualization of collaborating countries/regions by the number of publications. The radius of a node is made of three rings and reflects the number of publications, and the color indicates the average published year. High betweenness centrality indicates betweenness centrality of at least 0.1 and is represented by the thickness of the purple trim. (b) Overlay visualization of collaborating countries/regions by the number of citations. The size of the node represents the total citations, and the color represents the citations per publication. (c) Collaborating institutions network visualization. (d) The top 10 most productive institutions related to claudins in cancer research. Inserm, Institut National de la Sante et de la recherche Medicale. (e) Collaborating author network visualization. (f) Co-cited author network visualization.

The publications related to research on CLDNs in cancer covered 63 countries/regions (Figure 3), of which the number of publications published by China ranked first with 494 publications (28.72%), followed by the United States (383 publications; 22.27%), Japan (283 publications; 16.45%), Germany (131 publications; 7.62%) and South Korea (95 publications; 5.52%) (Supplementary Figure S1A). In most cases, countries with more published literature also have more citations. In contrast, although the number of relevant publications published by the United States (383 publications) is lower than that of China (494 publications), the citations of the United States (20464 citations) ranked first, far exceeding China (9389 citations), which is the second country in citations (Figure 4a,b).

Through the analysis of changes in the annual publications of the top five productive countries/regions (Supplementary Figure S1B), the overall trend in the annual publications of China showed a linear increase since 2009. On the other hand, the overall variations in the annual publications of the United States, Japan, and Germany were not significant. In 2005, China published only one relevant study. In 2010, the number of relevant articles published by China exceeded that of South Korea and Germany and was lower than that of the United States and Japan. Subsequently, the annual publications of China surpassed those of Japan and the United States in 2013 and 2014, respectively.

Among these 63 countries/regions, the United States ranked first with a betweenness centrality of up to 0.61, which was much higher than those of other countries/regions with a betweenness centrality of over 0.1 for Germany (0.20), Brazil (0.18), France (0.16), Japan (0.13), the United Kingdom (0.13), Sweden (0.12), and the Czech Republic (0.12) (Figure 4a). It is worth noting that although France, Sweden, and the Czech Republic did not rank among the top 10 productive countries/regions, these countries had a high betweenness centrality, playing an important hub connecting role in the cooperation of relevant studies. Unfortunately, China was the most productive country, but the betweenness centrality of China was only 0.07. Similarly, although South Korea, Canada, Italy, and Hungary ranked among the top 10 productive countries/regions, their betweenness centrality, similar to that of China, did not exceed 0.1. Obviously, the United States (35 links) had the closest cooperation with other countries/regions, and its most important partners were China, Germany, and Japan (Figure 4b; Supplementary Figure S1c, d). In recent years, countries/regions such as China, Turkey, Iran, India, Russia, and Romania have continuously increased their publications in this field (Supplementary Figure S1C), especially Iran, India, and Russia (Supplementary Table S1). Australia with 60.65 was the country with the most citations per publication, followed by the Netherlands (55.10 citations per publication) and the United States (53.43 citations per publication) (Figure 4b).

Through the analysis of changes in the annual publications of the top five productive countries/regions (Supplementary Figure S1b), the overall trend in the annual publications of China showed a linear increase since 2009. On the other hand, the overall variations in the annual publications of the United States, Japan, and Germany were not significant. In 2005, China published only one relevant study. In 2010, the number of relevant articles published by China exceeded that of South Korea and Germany and was lower than that of the United States and Japan. Subsequently, the annual publications of China surpassed those of Japan and the United States in 2013 and 2014, respectively.

The publications related to CLDNs in the field of cancer covered 1891 institutions. Both Harvard University (0.19) and the University of Texas System (0.17) had high betweenness centrality (Figure 4c). Figure 4d shows the top 10 most productive institutions ranked by the number of studies, including two institutions in China (China Medical University and Jilin University), four institutions in the United States (Harvard University, Johns Hopkins University, University of Texas System and National Institutes of Health), two institutions in Japan (Sapporo Medical University and Osaka University), one institution in Hungary (Semmelweis University) and one institution in France (Institut National de la Sante et de la Recherche Medicale). China Medical University was the most productive institution, with 63 publications, followed by Semmelweis University (48) and Sapporo Medical University (45). Johns Hopkins University had the most citations at 3044 and the highest H-Index of 29 in these institutions.

The publications related to CLDNs in the field of cancer covered 9921 authors, including 169 authors with at least 5 publications. Table 1 shows that Xue Yixue published 34 relevant studies and was the most productive author. Liu Yunhui and Sawada Norimasa shared the title of the second most productive author with 29 relevant studies published. Sawada Norimasa ranked first with 41.66 citations per publication, followed by Wang Ping with 34.15 citations per publication and Kojima Takashi with 32.92 citations per publication.

**Table 1.** The top 10 most productive authors.

Rank	Author	Publications	Citations	Citations/Publication
1	Xue Yixue	34	1019	29.97
2	Liu Yunhui	29	952	32.83
3	Sawada Norimasa	29	1208	41.66
4	Kojima Takashi	25	823	32.92
5	Schaff Zsuzsa	22	546	24.82
6	Ikari Akira	21	478	22.76
7	Kondoh Masuo	21	332	15.81
8	Quan Chengshi	20	429	21.45
9	Takasawa Akira	20	416	20.80
10	Wang Ping	20	683	34.15

None of the authors had a betweenness centrality over 0.1, indicating that there was no extensive collaboration among authors in this field (Figure 4e). Kondoh Masuo (Osaka University) and Quan Chengshi (Jilin University) belonged to the largest connected component containing 436 nodes. Some of the top 10 most productive authors had formed collaborative networks with their own institutions, such as China Medical University (Xue Yixue, Liu Yunhui, and Wang Ping) and Sapporo Medical University (Sawada Norimasa, Kojima Takashi, and Takasawa Akira). Schaff Zsuzsa (Semmelweis University) and Ikari Akira (Gifu Pharmaceutical University) did not have a collaborative network with the other top 10 most productive authors (Figure 4e).

Tsukita S was the most co-cited author with 423 citations, followed by Furuse M with 342 citations and Kominsky SL with 310 citations. Both Kominsky SL and Soini Y had a high betweenness centrality over 0.1 (Figure 4f). Seven clusters of co-cited author networks were obtained with a significant Q-value and reasonable weighted mean S-value (Q = 0.3375; S = 0.7566). The most active clusters were #0 ‘lung adenocarcinoma’ (S = 0.737; 62; 2016), #5 ‘gastric cancer’ (S = 0.919; 31; 2017) and #3 ‘claudin 1’ (S = 0.75; 39; 2011) (Supplementary Figure S2). Furthermore, the top three co-cited authors with the most important strength of burst were Siegel RL (42.76), Tabaries S (32.15) and Gunzel D (27.15) (Supplementary Table S2).

### Journals and references

**Figure 5.**
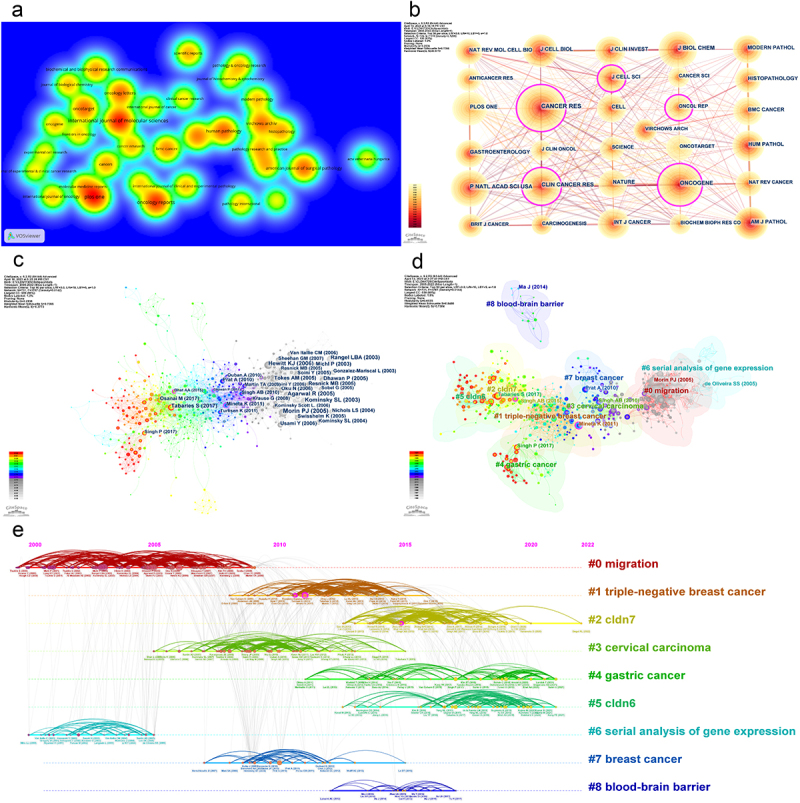
Source journals, co-cited journals, and co-cited references visualization. (a) Density visualization of the source journals. (b) Co-cited journal network. (c) Co-cited reference network. (d) Co-cited reference clusters. (e) Timeline visualization of co-cited references during 2005–2022.

*PLoS One*, with 43 publications, was the most productive journal of 552 different sources related to research on CLDNs in cancer, followed by the *International Journal of Molecular Sciences* (42 publications) and *Oncology Reports* (37 publications) (Figure 5a). Among the top 10 productive journals, *PLoS One* had the highest citations at 1,713, the *American Journal of Surgical Pathology* had the highest Journal Impact Factor (JIF) at 6.298, and *BMC Cancer* had the highest citations per publication at 47.68 (Supplementary Table S3). The top three most co-cited journals were *Cancer Research* (1136 citations), *Oncogene* (887 citations) and *Clinical Cancer Research* (782 citations). The co-cited journals with a betweenness centrality of at least 0.1 are *Cancer Research* (0.17), *Oncogene* (0.15), *Clinical Cancer Research* (0.12), *Journal of Cell Science* (0.11), and *Oncology Reports* (0.10) (Figure 5b).

The top 10 cited publications based on frequency are listed in Table 2. ‘*A SNAIL1-SMAD3/4 transcriptional repressor complex promotes TGF-beta mediated epithelial – mesenchymal transition*’ [28] published in *Nature Cell Biology* was the most cited publication with 499 citations, followed by ‘*Claudin proteins in human cancer: promising new targets for diagnosis and therapy*’ [13] published in *Cancer Research* with 439 citations and ‘*Proteomics analysis of A33 immunoaffinity-purified exosomes released from the human colon tumor cell line LIM1215 reveals a tissue-specific protein signature*’ [29] published in *Molecular & Cellular Proteomics* with 431 citations.

**Table 2.** The top 10 most cited publications.

Rank	Publication Title	Journal	Citations	JIF2021	Quartile
1	A SNAIL1-SMAD3/4 transcriptional repressor complex promotes TGF-beta mediated epithelial – mesenchymal transition [28]	Nature Cell Biology	499	28.213	Q1
2	Claudin proteins in human cancer: promising new targets for diagnosis and therapy [13]	Cancer Research	439	13.312	Q1
3	Proteomics analysis of A33 immunoaffinity-purified exosomes released from the human colon tumor cell line LIM1215 reveals a tissue-specific protein signature [29]	Molecular & Cellular Proteomics	431	7.381	Q1
4	Tight junctions and the modulation of barrier function in disease [87]	Histochemistry and Cell Biology	428	2.531	Q2
5	Epithelial cell adhesion molecule – More than a carcinoma marker and adhesion molecule [88]	American Journal of Pathology	418	5.770	Q1
6	Claudin-1 regulates cellular transformation and metastatic behavior in colon cancer [89]	Journal of Clinical Investigation	416	19.477	Q1
7	Epithelial – mesenchymal transition in tumor metastasis [90]	Molecular Oncology	412	7.449	Q1
8	Epithelial barriers in homeostasis and disease [91]	Annual Review of Pathology-Mechanisms of Disease	404	32.375	Q1
9	The claudin gene family: expression in normal and neoplastic tissues [14]	BMC Cancer	377	4.638	Q2
10	Loss of tight junction barrier function and its role in cancer metastasis [92]	Biochimica et Biophysica Acta – Biomembranes	325	4.019	Q3

The most frequently co-cited reference was ‘*Claudin proteins in human cancer: promising new targets for diagnosis and therapy*’ [13], with 93 co-citations published in *Cancer Research*, and this literature was also the second most frequently cited publication related to research on CLDNs in cancer (Figure 5c; Supplementary Table S4). The second and third most co-cited references based on frequency were ‘*The claudin gene family: expression in normal and neoplastic tissues*’ [14] published in *BMC Cancer* and ‘*Claudin-3 and claudin-4 expression in ovarian epithelial cells enhances invasion and is associated with increased matrix metalloproteinase-2 activity*’ [30] published in *Cancer Research*, with 85 and 84 co-citations, respectively. Three co-cited references with high betweenness centrality were Bhat et al., 2015 [31] (0.16), Dhawan et al. [32] (0.12), and Mineta et al. [33] (0.10). Coincidentally, the top 10 co-cited references with the most important strength of burst could be found among the top 10 co-cited frequency references and three co-cited references with high betweenness centrality (Table 3). ‘*Predicted expansion of the claudin multigene family*’ (∑ = 8.04) [33] was the most influential potential co-cited reference (Supplementary Table S5).

**Table 3.** The top 10 co-cited references with the strongest citation bursts.

Rank	Co-cited Reference	Year	Strength	Begin	End	2005–2022
1	Loss of the tight junction protein claudin-7 correlates with histological grade in both ductal carcinoma in situ and invasive ductal carcinoma of the breast [48]	2003	37.17	2005	2008	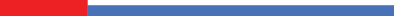
2	Tight junction proteins claudin-3 and claudin-4 are frequently overexpressed in ovarian cancer but not in ovarian cystadenomas [93]	2003	31.26	2005	2008	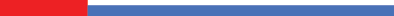
3	Claudin-4 expression decreases invasiveness and metastatic potential of pancreatic cancer [55]	2003	26.28	2005	2008	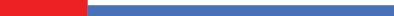
4	Claudin-3 and claudin-4 expression in ovarian epithelial cells enhances invasion and is associated with increased matrix metalloproteinase-2 activity [30]	2005	31.28	2006	2010	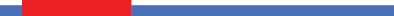
5	Claudin-1 regulates cellular transformation and metastatic behavior in colon cancer [89]	2005	26.37	2006	2010	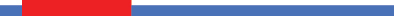
6	Claudin proteins in human cancer: promising new targets for diagnosis and therapy [13]	2005	35.64	2007	2010	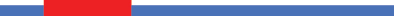
7	The claudin gene family: expression in normal and neoplastic tissues [14]	2006	30.3	2007	2011	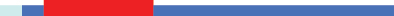
8	Predicted expansion of the claudin multigene family [33]	2011	21.81	2012	2016	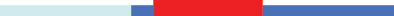
9	The role of claudins in cancer metastasis [66]	2017	23.33	2018	2022	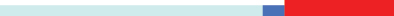
10	Cancer statistics, 2018 [94]	2018	27.28	2020	2022	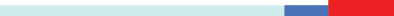

According to authors’ keywords, a total of nine clusters were obtained with a significant modularity Q-value (Q = 0.6535) and a convincing weighted mean S-value (S = 0.8488) (Figure 5d). These clusters represented the major research trends on claudins in the field of cancer. According to Figure 5e and Supplementary Figure S2, the trends could be divided into three periods, the early period (before 2008), middle period (from 2009 to 2014), and later period (2015 to present). Clusters #6 ‘serial analysis of gene expression’ (S = 0.923; 2003) and #0 ‘migration’ (S = 0.822; 2005) were relatively early hotspots (2005–2008). Since 2009, the trend has gradually evolved into clusters #3 ‘cervical carcinoma’ (S = 0.851; 2009) and #7 ‘breast cancer’ (S = 0.955; 2010) and then continued to evolve into clusters #1 ‘triple-negative breast cancer’ (S = 0.719; 2012) and #4 ‘gastric cancer’ (S = 0.794; 2016). This research trend has continued to develop to clusters #2 ‘CLDN7’ (S = 0.854; 2016) and #5 ‘CLDN6’ (S = 0.923; 2017) since 2016. Clusters #3 ‘cervical carcinoma’ (S = 0.851; 2009) and #7 ‘breast cancer’ (S = 0.955; 2010) disappeared gradually and vanished in 2018. Furthermore, a minor isolated cluster #8 ‘blood‒brain barrier’ (S = 0.998; 2015) appeared in 2016 and then disappeared in 2019. The trend focused on three clusters, #2 ‘CLDN7’ (S = 0.854; 2016), #4 ‘gastric cancer’ (S = 0.794; 2016) and #5 ‘CLDN6’ (S = 0.923; 2017), until 2022 (Figure 5e; Supplementary Figure S3).

### Authors’ keywords

**Figure 6.**
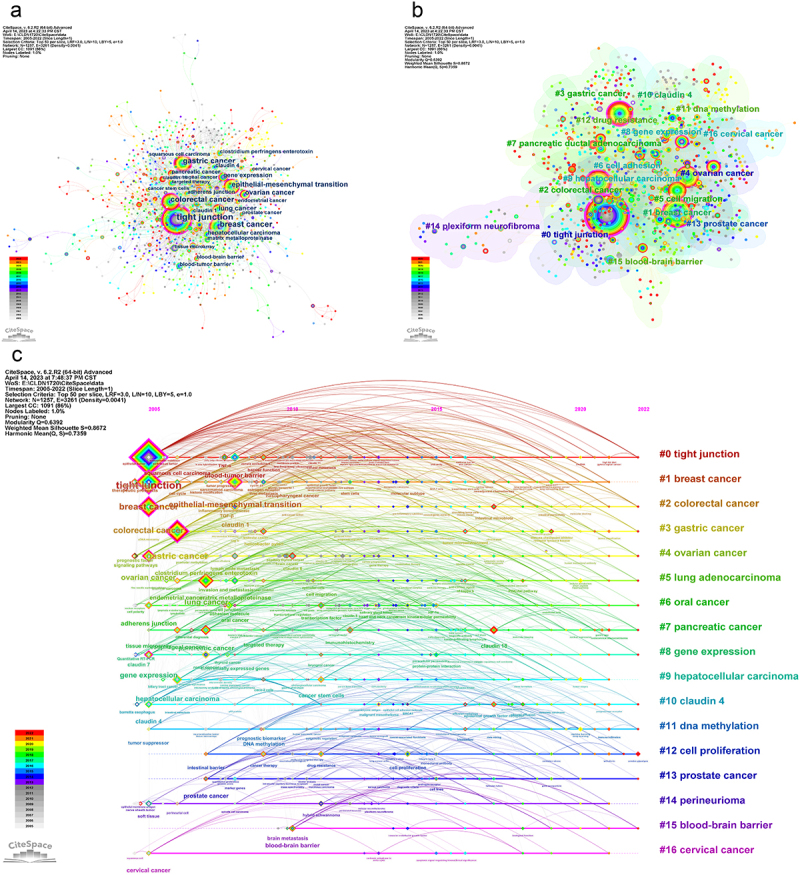
Co-occurrence of authors’ keywords. (a) Keyword co-occurrence networks. (b) Keyword co-occurrence clusters. (c) Timeline visualization of keywords.

A total of 1257 nodes and 2864 connections of the authors’ keyword co-occurrence network are displayed in Figure 6a after merging different forms of the same keyword (such as CLDN1, CLDN-1, and claudin-1 merging into claudin1) and different subtypes of the same cancer (such as lung adenocarcinoma and non-small cell lung cancer merging into lung cancer). Authors’ keywords with a high frequency of occurrence and high betweenness centrality were ‘tight junction’ (284; 0.55), ‘breast cancer’ (143; 0.25), ‘gastric cancer’ (117; 0.23), ‘colorectal cancer’ (113; 0.21), ‘epithelial – mesenchymal transition’ (EMT) (88; 0.17), ‘ovarian cancer’ (65; 0.12), and ‘gene expression’ (43; 0.11).

Among the keywords with a frequency of occurrence of at least 10 times, ‘tight junction’, ‘adherens junction’, ‘claudin 1’, ‘clostridium perfringens enterotoxin’, ‘claudin 4’, ‘matrix metalloproteinase (MMP)’, ‘claudin 18’, ‘claudin 7’, ‘TGF-β’, and ‘tumour suppressor’ were classified as molecules. ‘EMT’, ‘gene expression’, ‘blood–tumour barrier’, ‘blood‒brain barrier’, ‘brain metastasis’, ‘DNA methylation’, ‘cell proliferation’, ‘intestinal barrier’, ‘differentially expressed genes’, and ‘invasion and metastasis’ were classified as phenotypes and structures. The top 10 most frequent cancer keywords were ‘breast cancer’, ‘gastric cancer’, ‘colorectal cancer’, ‘ovarian cancer’, ‘lung cancer’, ‘hepatocellular carcinoma’, ‘pancreatic cancer’, ‘prostate cancer’, ‘oesophageal cancer’, and ‘cervical cancer’ (Table 4). Furthermore, ‘tissue microarray’ (16) and ‘targeted therapy’ (15), two technical keywords, are also shown in Figure 6a.

**Table 4.** The co-occurrence statistics of major authors’ keywords.

Molecule	Count	Cancer	Count	Phenotype and Structure	Count
tight junction	284	breast cancer	143	epithelial – mesenchymal transition	88
adherens junction	28	gastric cancer	117	gene expression	43
claudin 1	27	colorectal cancer	113	blood – tumor barrier	26
clostridium perfringens enterotoxin	25	ovarian cancer	65	blood‒brain barrier	21
claudin 4	25	lung cancer	61	brain metastasis	14
matrix metalloproteinase	24	hepatocellular carcinoma	34	DNA methylation	13
claudin 18	19	pancreatic cancer	33	cell proliferation	12
claudin 7	14	prostate cancer	22	intestinal barrier	11
TGF-β*	12	oesophageal cancer	22	differentially expressed genes	11
tumour suppressor	12	cervical cancer	21	invasion and metastasis	10

*TGF-β, transforming growth factor-beta.

Figure 6b shows 17 clusters extracted with a significant modularity (Q = 0.6392) and a credible weighted mean silhouette score (S = 0.8672): #0 ‘tight junction’ (S = 0.853; 117; 2014), #1 ‘breast cancer’ (S = 0.828; 105; 2013), #2 ‘colorectal cancer’ (S = 0.886; 82; 2016), #3 ‘gastric cancer’ (S = 0.776; 81; 2015), #4 ‘ovarian cancer’ (S = 0.841; 81; 2014), #5 ‘lung adenocarcinoma’ (S = 0.849; 79; 2015), #6 ‘oral cancer’ (S = 0.885; 74; 2012), #7 ‘pancreatic cancer’ (S = 0.905; 73; 2015), #8 ‘gene expression’ (S = 0.816; 67; 2013), #9 ‘hepatocellular carcinoma’ (S = 0.829; 57; 2015), #10 ‘claudin 4’ (S = 0.928; 54; 2014), #11 ‘DNA methylation’ (S = 0.881; 44; 2016), #12 ‘cell proliferation’ (S = 0.888; 42; 2017), #13 ‘prostate cancer’ (S = 0.885; 40; 2013), #14 ‘perineurioma’ (S = 0.977; 32; 2013), #15 ‘blood‒brain barrier’ (S = 0.963; 26; 2018) and #16 ‘cervical cancer’ (S = 0.983; 17; 2015). Clusters #1, #9, #14, and #16 were less active in 2022 than other clusters (Figure 6c).

The top 20 keywords with the strongest co-occurrence bursts are listed in Table 5. The most prominent word was ‘tight junction’ with a strength of 10.02, followed by ‘gene expression’ (6.72) and ‘claudin 1’ (5.94). Overall, the beginning of keyword co-occurrence bursts could be divided into two phases, the early to middle period (before 2015), ‘tight junction’, ‘gene expression’, ‘ovarian cancer’, ‘matrix metalloproteinase’, ‘tissue microarray’, ‘blood–tumour barrier’, ‘low-frequency ultrasound’, ‘hybrid schwannoma’, ‘head and neck cancer’, ‘claudin 1’, beginning to burst; and the middle to later period (2015 and beyond), ‘protein‒protein interaction’, ‘HIF-1α’, ‘cervical cancer’, ‘EMT’, ‘lung cancer’, ‘claudin 18’, ‘sarcomatoid carcinoma’, ‘oesophageal cancer’, ‘intestinal microbiota’, and ‘intestinal barrier’ beginning to burst. Additionally, ‘claudin 18’, ‘intestinal microbiota’, and ‘intestinal barrier’ are still in the research spotlight until 2022.

**Table 5.** The top 20 authors’ keywords with the strongest co-occurrence bursts.

Rank	Keyword	Year	Strength	Begin	End	2005–2022
1	tight junction	2005	10.02	2005	2007	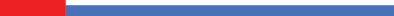
2	gene expression	2005	6.72	2005	2009	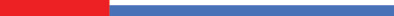
3	ovarian cancer	2005	3.13	2006	2013	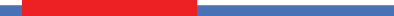
4	matrix metalloproteinase	2008	3.63	2009	2013	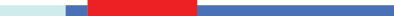
5	tissue microarray	2005	2.64	2009	2013	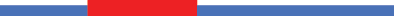
6	blood – tumor barrier	2008	4.61	2010	2015	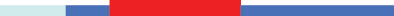
7	low-frequency ultrasound	2010	2.41	2010	2011	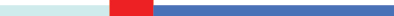
8	hybrid schwannoma	2011	2.27	2011	2015	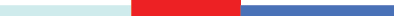
9	head and neck cancer	2013	2.32	2013	2017	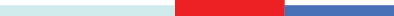
10	claudin 1	2008	5.94	2014	2015	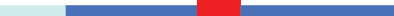
11	protein‒protein interaction	2015	2.59	2015	2018	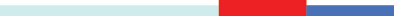
12	HIF-1α*	2015	2.46	2015	2017	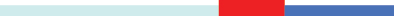
13	cervical cancer	2005	2.31	2015	2016	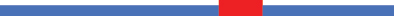
14	epithelial – mesenchymal transition	2008	4.22	2017	2019	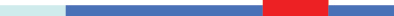
15	lung cancer	2007	2.61	2018	2018	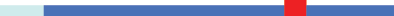
16	claudin 18	2017	4.96	2019	2022	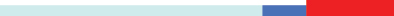
17	sarcomatoid carcinoma	2008	2.38	2019	2020	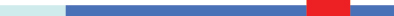
18	oesophageal cancer	2006	3.00	2020	2020	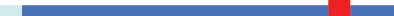
19	intestinal microbiota	2017	2.91	2021	2022	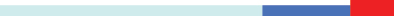
20	intestinal barrier	2007	2.89	2021	2022	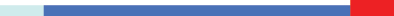

*HIF-1α, hypoxia inducible factor-1 alpha.

## Discussion

### Contributions to research on claudins in cancer

A total of 1720 publications on CLDNs in the field of cancer from 2005 to 2022 were included in this study. These publications cited 32,328 articles and were published by 552 different sources, covering 63 countries/regions, 1891 institutions, and 9921 authors. The annual publications and citations of these publications increased continuously, with the linear equation Publications = 6.1321 Year + 37. 301, R^2^ = 0.9162 and Citations = 362.79 Year − 579.95, R^2^ = 0.946, respectively. Thus, research on CLDNs in the field of cancer has received increasing attention. China, the United States, Japan, Germany, and South Korea are the top five productive countries, with 1255 papers, accounting for 72.97% of the total, although some of them were published not alone but in collaboration with other countries. Annual publications in the United States, Japan, Germany, and South Korea did not increase linearly, as China showed. This is perhaps the reason why the number of articles published per year in the world increased linearly rather than exponentially. This finding also suggests that research on CLDNs in cancer may still have considerable vitality, and there may be much room for exploration in the future.

China was the most productive country, without a high betweenness centrality and a strong burstness. Similarly, China Medical University, Semmelweis University, and Sapporo Medical University were the most productive institutions, but their citations were less than those of Johns Hopkins University, Harvard University, and the National Institutes of Health. The United States had the most citations, most extensive cooperative network, highest betweenness centrality, and strongest burstness and was the second most productive country. Although the research institutions in the United States did not rank among the top three productive institutions, there were four institutions in the United States (Harvard University, Johns Hopkins University, University of Texas System, and National Institutes of Health) among the top 10 productive institutions. Johns Hopkins University had the most citations and highest betweenness. Seven publications were published by the United States among the top 10 of both cited publications and co-cited references. The corresponding author of three of these top co-cited references based on frequency was Morin PJ of the National Institute on Aging (NIA). According to the above data, the United States dominates the literature research on CLDNs in cancer, and China is also becoming an increasingly important contributor.

Research institutions have many published and cited articles, which are usually contributed by authors with high output and high citations [20]. Among the top 10 productive authors, Kondoh Masuo (Osaka University) and Quan Chengshi (Jilin University) belonged to the largest connected component containing 436 authors covering only 4.38% of the overall authors. Some of the top 10 most productive authors had formed collaborative networks with their own institutions, such as China Medical University (Xue Yixue, Liu Yunhui, and Wang Ping) and Sapporo Medical University (Sawada Norimasa, Kojima Takashi, and Takasawa Akira). Schaff Zsuzsa (Semmelweis University) and Ikari Akira (Gifu Pharmaceutical University) did not have a collaboration network with the other top 10 most productive authors. These results indicate the need to strengthen the communication and cooperation among different institutions to promote more progress in relevant research.

### Trends and hotspots of claudins in cancer research

In 1998, CLDN1 and CLDN2 localized at TJs were first isolated from chicken liver by Shoichiro Tsukita’s research group at Kyoto University [10]. Thereafter, this research group reported additional CDLNs designated CLDN3 to CLDN15 [34–36]. CLDN16 was identified by Simon [37] of Yale University as paracellin-1 (PCLN1), which is required for paracellular Mg^2+^ resorption. Increasing evidence suggests that CLDNs, as key components of TJs, can interact directly with ZO-1, ZO-2, ZO-3, and the actin cytoskeleton to perform barrier and paracellular permeability functions [38–41].

Aware of the important role of CLDNs [42,43], researchers began to study CLDNs in the field of cancer. The impact of CLDNs on cancer progression and migration, as well as their expression in different cancers, was the first to be understood. Early research on CLDNs in cancer showed that CLDN1 was expressed at low levels in breast cancer [44,45] and glioblastoma multiforme [46] but was highly expressed in primary colorectal cancer [47]. CLDN5 was significantly downregulated in glioblastoma multiforme [46]. CLDN7 expression is downregulated in breast invasive ductal carcinoma, especially in high-grade lesions [48]. The clustering results of co-cited references also showed that the early period studies focused on clusters #0 ‘migration’ and #6 ‘serial analysis of gene expression’ (Figure 5e; Supplementary Figure S3). The article that reported CLDN1 as having an important role in the invasion and metastasis of breast cancer was the most influential potential co-cited reference of cluster migration [49]. Simultaneously, the analysis of the expression of multiple genes has been achieved due to the rapid development of sequencing technology. A study first used SAGE from various ovarian cell lines and tissues and found that CLDN3 and CLDN4 were overexpressed in ovarian cancer [50]. These results, published in *Cancer Research* by a team of researchers led by Morin PJ of the NIA, have received much attention. The first author of this article was Hough CD, who became one of the top 10 co-cited authors with the strongest citation bursts in research on CLDNs in cancer during 2005–2022 (Supplementary Table S2). The important role of CLDN1 in the skin barrier function of mice [51] and the low expression of CLDN4 in advanced gastric adenocarcinoma were also found [52].

During the middle period of research on CLDNs in cancer, #7 ‘breast cancer’, #1 ‘triple-negative breast cancer’, #3 ‘cervical carcinoma’, and a minor isolated cluster #8 ‘blood‒brain barrier’ received attention. Thanks to the application of omics techniques, the expression of various CLDNs in breast cancer has been detected in cell lines, animal models, and pathological tissues. Due to the heterogeneity of tumors, a ‘claudin-low’ subtype of breast cancer was identified, characterized by low expression of the TJ proteins CLDN3, CLDN4, and CLDN7, with a poor prognosis and features of mesenchymal and mammary stem cells [53]. During this period, three new CLDN members were identified [33], and a total of 26 human CLDN members were discovered [11]. The expression distribution of CLDNs in different tissues and their impact on systemic development and genetic diseases have also been well summarized and elaborated [11]. With attention given to the biological function of long noncoding RNA (lncRNA) regulating gene expression, lncRNA TUG1 regulating CLDN5 expression to influence blood – tumor barrier permeability has been reported [54].

To better grasp the change in research trends and hotspots, timeline visualizations of co-cited authors and authors’ keywords were analyzed, as well as authors’ keywords with the strongest co-occurrence bursts analyzed in this study. Because hotspots do not suddenly appear or disappear, the period of research on CLDNs in cancer was divided into an early-middle period (before 2015) and a later period (2015 and beyond) based on clustering labels and authors’ keywords (including co-occurrence and bursts).

During the early-middle period, TJs were the most mentioned biological structures, and several molecules received attention, such as ZO-1, CLDN1, CLDN4, Clostridium perfringens enterotoxin (CPE), and matrix metalloproteinase (MMP). Breast cancer, ovarian cancer, prostate cancer, hybrid schwannoma, head and neck cancer, perineurioma, and oral cancer were the most commonly related to CLDN research, especially breast cancer, ovarian cancer, and prostate cancer. Gene expression and tissue microarray were the most commonly mentioned technologies. TJ and ZO-1 were inevitably mentioned when CLDN was mentioned because CLDN could interact directly with ZO-1 to constitute a TJ [38,39,41]. Moreover, CLDN1 and CLDN4 were the CLDN members involved earlier in cancer studies [44,46,50,55]. CPE, a naturally occurring single polypeptide of 35 kDa, can cause several common gastrointestinal diseases and is a naturally occurring CLDN3 and CLDN4 inhibitor that binds to the second extracellular loop [56]. CLDN4 was also known as CPE receptor (CPE-R) before the discovery of CLDN [34]. The COOH-terminal fragment of CPE (C-CPE) retains receptor-binding activity without the toxicity of enterotoxin [57]. Thus, both CPE and C-CPE could be used as diagnostic tools and in early screening of breast, ovarian, or pancreatic cancers [58,59]. Additionally, C-CPE can carry various cytotoxic drugs or molecules, such as TNF, to induce the death of cancer cells expressing CLDN3 or CLDN4 [60,61]. MMPs are a multigene family that plays a remarkable number of regulatory roles in cancer progression, such as angiogenesis, invasion, and metastasis [62]. Many studies have confirmed that CLDNs could regulate various MMPs to influence tumor progression [63], such as CLDN3 and CLDN4, indicating that MMP2 increases invasion and metastasis in ovarian cancer [30]. During the early-middle period, tissue microarray and immunohistochemistry were used to detect gene expression of the abovementioned CLDN members in various cancers [15,64,65].

For the later period, clusters #5 ‘CLDN6’, #2 ‘CLDN7’, and #4 ‘gastric cancer’ of co-cited references received the most attention. Gastric cancer, colorectal cancer, lung cancer (especially lung adenocarcinoma), hepatocellular carcinoma, pancreatic cancer, esophageal cancer, cervical cancer, and sarcomatoid carcinoma received the most attention during the later period of research on CLDNs in cancer. CLDN18 and HIF-1α were the most high-profile molecules. Many biological structures and phenotypes have also been noted by researchers, such as the intestinal barrier, intestinal microbiota, cell proliferation, EMT, DNA methylation, and protein‒protein interactions. Many studies have shown a complex relationship between CLDN expression and tumor prognosis because CLDNs can either promote tumor development or inhibit tumor progression [66]. For example, CLDN7 has a dual role in colorectal cancer [31,67]. There is a possibility that CLDN7 might be lost early during colorectal cancer progression but regained in locally invasive or disseminated colorectal cancer cells [66]. Targeted therapy and immunotherapy have received increasing attention in the treatment of various cancers [68]. Due to the high specific expression of CLDN18 in the stomach and lung (Supplementary Figure S4), the treatment of gastric cancer or gastroesophageal junction cancer targeting CLDN18 has received increasing attention, such as the anti-CLDN chimeric monoclonal antibodies claudiximab and zolbetuximab and anti-CLDN18.2 chimeric antigen receptor (CAR)-T-cell immunotherapy [68–72]. CLDN18.2 CAR-T cells therapy was clinically shown to be effective in patients with gastric cancer, resulting in an objective response rate (ORR) of 57.1% and a disease control rate (DCR) of 75.0% [73]. CLDN6 was also used as a CAR target in a solid tumor therapy model [74], and the clinical results showed that CLDN6 CAR-T cells therapy in solid tumor with an ORR of 33.3% and a DCR of 66.7% [75].

According to the change of authors’ keywords popularity, EMT was received increasing attention. CLDNs is considered closely related to EMT that is a key process experienced by tumor cell metastasis [3,76]. EMT is regulated by multiple transcription factors and signaling pathways, such as TGF-β and HIF-1α [3]. TGF-β could induce the expression of CLDN3 and CLDN4 to enhance the EMT process of tumor cells [77,78]. Under hypoxic conditions, HIF-1α can upregulate CLDN6 transcription levels, and CLDN6 deletion may lead to HIF-1α-driven breast cancer metastasis [79]. Similarly, CLDN1 and CLDN4 also had a feedback regulatory effect on HIF-1α expression in esophageal squamous cell carcinoma [80]. Intestinal microbiota imbalance has been demonstrated to affect the expression of CLDN1 and to induce diseases from inflammatory bowel disease to colon cancer [81]. Various CLDNs are regulated by PI3K/AKT/mTOR signaling and play a role in tumor proliferation, invasion, and migration, such as CLDN1 in breast cancer [82], CLDN2 in renal clear cell carcinoma [83] and lung adenocarcinoma [84], CLDN3 in colorectal cancer [85] and lung adenocarcinoma [86], and CLDN4 in gastric cancer [87]. DNA methylation is considered an important way to regulate CLDN expression and influence cancer malignant progression. DNA hypomethylation is related to the transcriptional upregulation of CLDN4 in urinary bladder cancer [88] and CLDN3 in ovarian cancer [89]. Silencing of CLDN7 expression was confirmed to correlated with promoter hypermethylation [90]. However, DNA hypermethylation of the CLDN1 promoter promoted CLDN1 expression and inhibited the malignant progression of lung cancer [5]. In addition, research on CLDN12 in cancers was previously less commonly conducted because CLDN12 was considered a constitutive protein widely distributed in different tissues and organs [14]. We searched for mRNA expression data of 20 CLDN family members in different organ tumors and found that the transcriptional expression of *CLDNs* had tissue specificity and varied considerably in cancer tissues (unpublished study) when the Oncomine database was still providing services (Supplementary Figure S5). Interestingly, this result showed that CLDN12 mRNA was highly expressed in most cancers, such as lung cancer, colorectal cancer, and other cancers. A recent study confirmed that the homologous interactions mediated by CLDN12 were necessary for myeloid-derived suppressor cells (MDSCs) across endothelial cells into tumor tissue. The loss of mouse CLDN12 inhibited the migration of MDSCs into tumors, the growth and angiogenesis of transplanted tumors, and increased antitumour immune responses [91]. CLDN12 had the ability to promote cell migration in A549 cells and osteosarcoma cells, suggesting that CLDN12 might be a suitable diagnostic biomarker and therapeutic target [92–94]. Currently, there is an increasing exploration and discovery of the regulation and functional roles of CLDNs in various types of cancers. However, due to the heterogeneity of tumors, as well as the complex characteristics of the EMT process and the tumor microenvironment. It is imperative that future research focuses on conducting more detailed investigations to elucidate the influence of CLDNs on cancer progression. Such studies will provide invaluable information essential for guiding future therapeutic interventions.

Some limitations should be considered in this study. First, we only searched through SCI-Expanded because SCI-Expanded is a relatively authoritative publication source and represents a certain quality of articles. SCI-Expanded only provides full reference text and citation lists from 2005 to the present, so the publication period was limited to 2005–2022. Second, we also eliminated some articles that we thought were irrelevant by reading titles and abstracts, which may also introduce a certain degree of artificial bias. We admit that we did not cover all research publications on CLDNs in cancer. Considering that some recent publications have not been fully cited, research trends in the past two or 3 years may be limited. Finally, the citations are influenced by open access status, source journal, and other factors.

## Conclusions

This study is the first research on the trends and hotspots of CLDNs in the field of cancer through scientometric analysis and visualization. The study of CLDNs involved cancers in at least 15 different organs, of which the most interesting were breast cancer, ovarian cancer, prostate cancer during the early-middle period of research and gastric cancer, colorectal cancer, and lung cancer during the later period of research. Morin PJ of the NIA is credited with initiating research on CLDNs in cancer. The United States dominates research on CLDNs in cancer, and China is also becoming an increasingly important contributor. China Medical University is the most productive, and Johns Hopkins University has the most citations. Xue Yixue of China Medical University is the most productive author, and Tsukita S is the most co-cited author. The most frequently co-cited reference is ‘*Claudin proteins in human cancer: promising new targets for diagnosis and therapy*’, published in *Cancer Research*, which is the most co-cited journal. Due to the structure and function of CLDNs, the study of CLDNs has been closely related to EMT from the beginning. From the initial detection of differences in the expression of different CLDNs in tumor cases, the complex relationship and regulatory mechanisms between CLDNs and tumors continue to deepen. Tumor therapy targeting CLDNs has also shifted from anti-CLDN chimeric monoclonal antibodies to CAR-T-cell immunotherapy. Studies on CLDN members that were previously of less concern have gradually increased, such as CLDN18 and CLDN12. CLDN18, the intestinal barrier, and the intestinal microbiota are hotspots in this field. The functional importance of many CLDNs in cancer progression has been confirmed, and CLDNs have also become important diagnostic and prognostic biomarkers. However, the mechanism of CLDN-mediated metastasis still needs further investigation, and the development of CLDN-targeted therapies also needs to be explored in the future.

## Supplementary Material

Supplementary_Figures_and_Tables (1).docx

## Data Availability

The dataset about literature search records (including full records and cited references) analyzed in this work is publicly available at: https://data.mendeley.com/datasets/kb7cbyks5v/1.
