# Supplementary material for: The research trends and hotspots of claudins in the field of cancer
Source: Cell Adh Migr. 2025 Jun 20;19(1):2520641. doi: 10.1080/19336918.2025.2520641 (PMC12915401; doi:10.1080/19336918.2025.2520641)
Supplement: Supplementary_Figures_and_Tables (1).docx [file KCAM_A_2520641_SM9093.docx]

**Supplementary Figures and Tables**


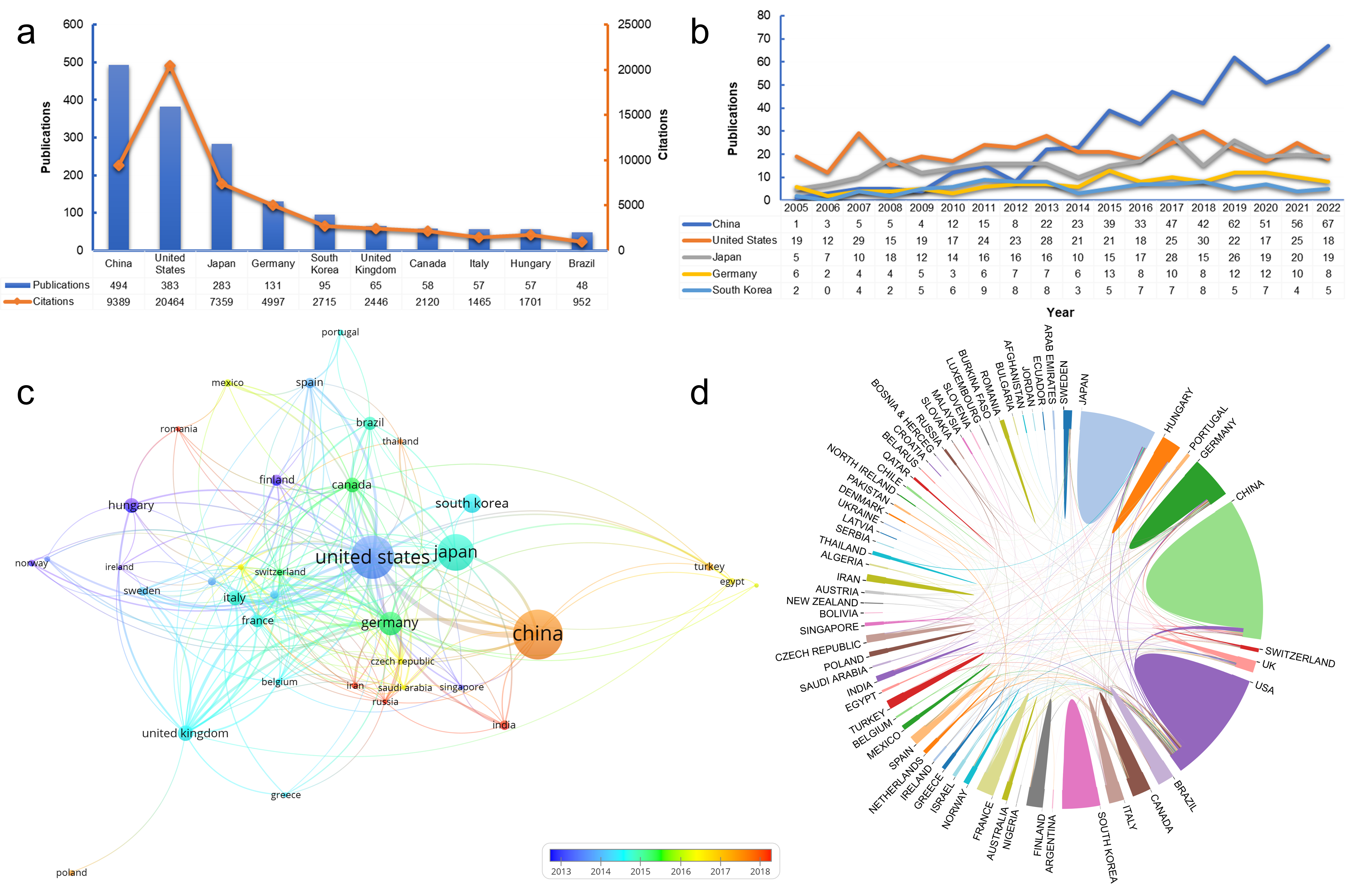


**Supplementary Figure S1**. Publications, citations and collaboration of countries/regions. (a) Publications and citations of the top 10 productive countries/regions. (b) Annual publications of the top 5 productive countries/regions. (c) Overlay visualization of collaborating countries/regions by the number of publications. The size of the node represents the number of relevant publications, and the color represents the average year of relevant literature published. (d) Collaborating countries/regions analysis. The size of the color block represents the number of publications.


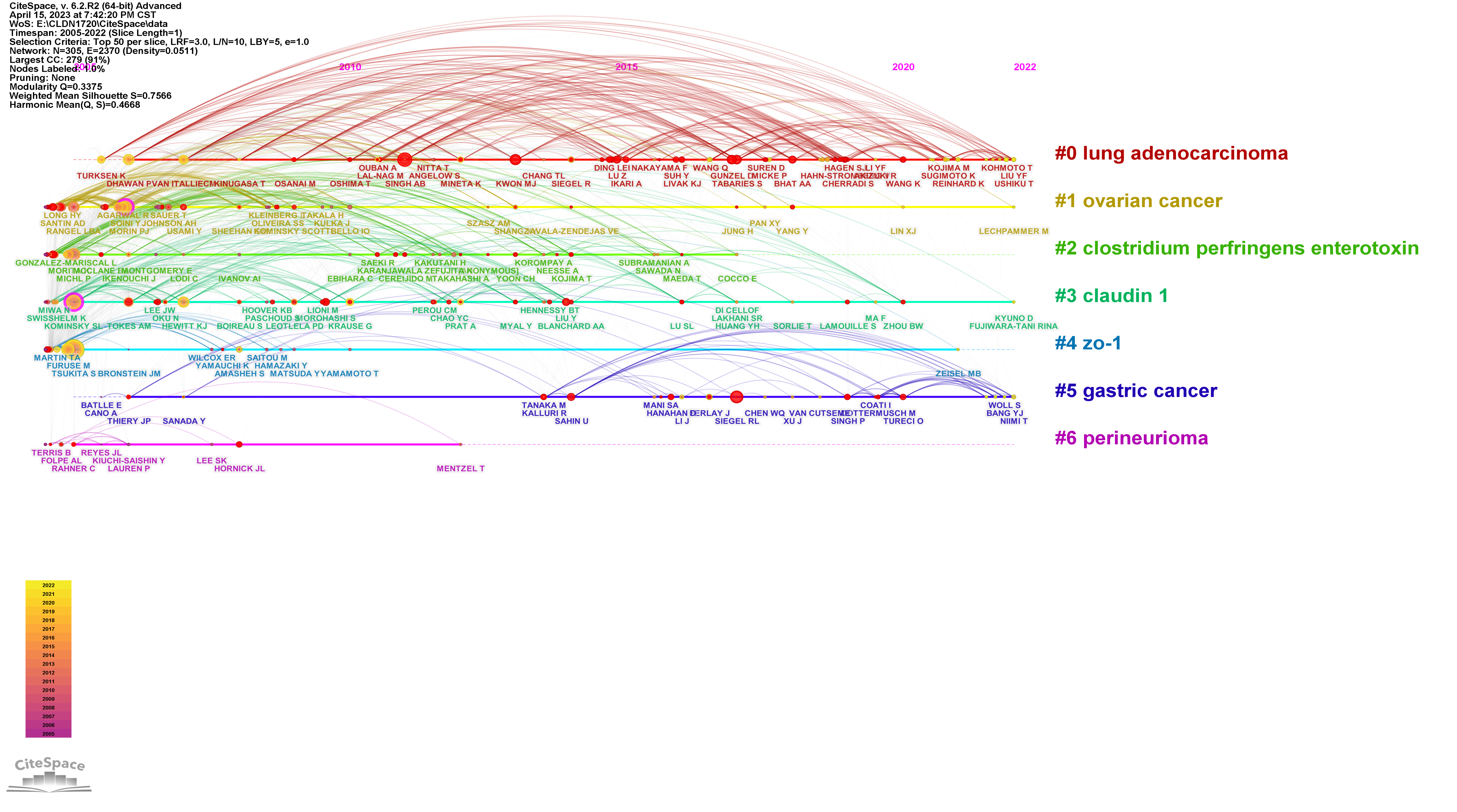


**Supplementary Figure S2**. Timeline visualization of co-cited authors.


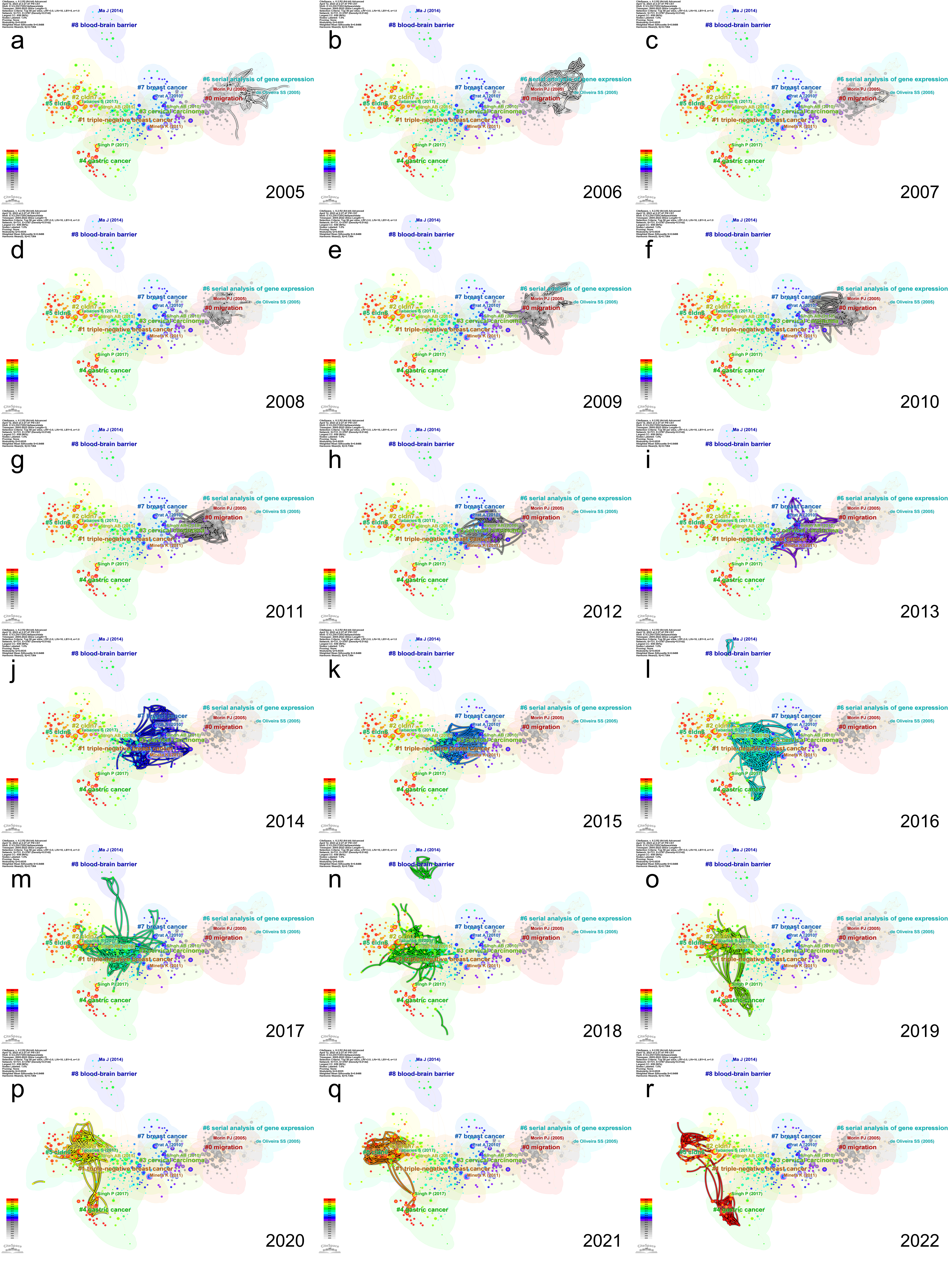


**Supplementary Figure S3**. The link walkthrough among co-cited reference clusters during 2005-2022.


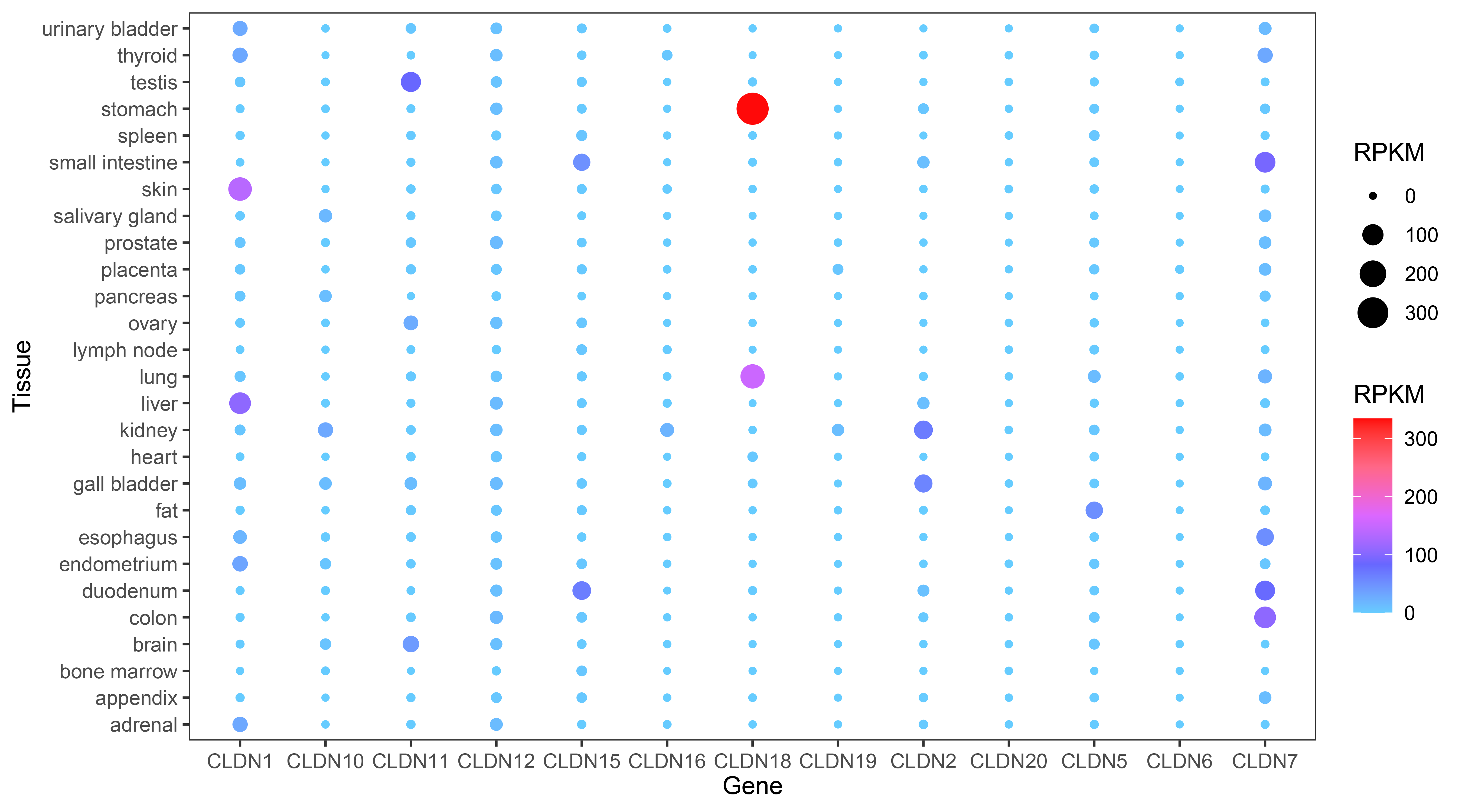


**Supplementary Figure S4**. Transcriptional expression of claudins (*CLDNs*) in 27 different normal tissues (NCBI). RPKM, Reads Per Kilobase per Million mapped reads.


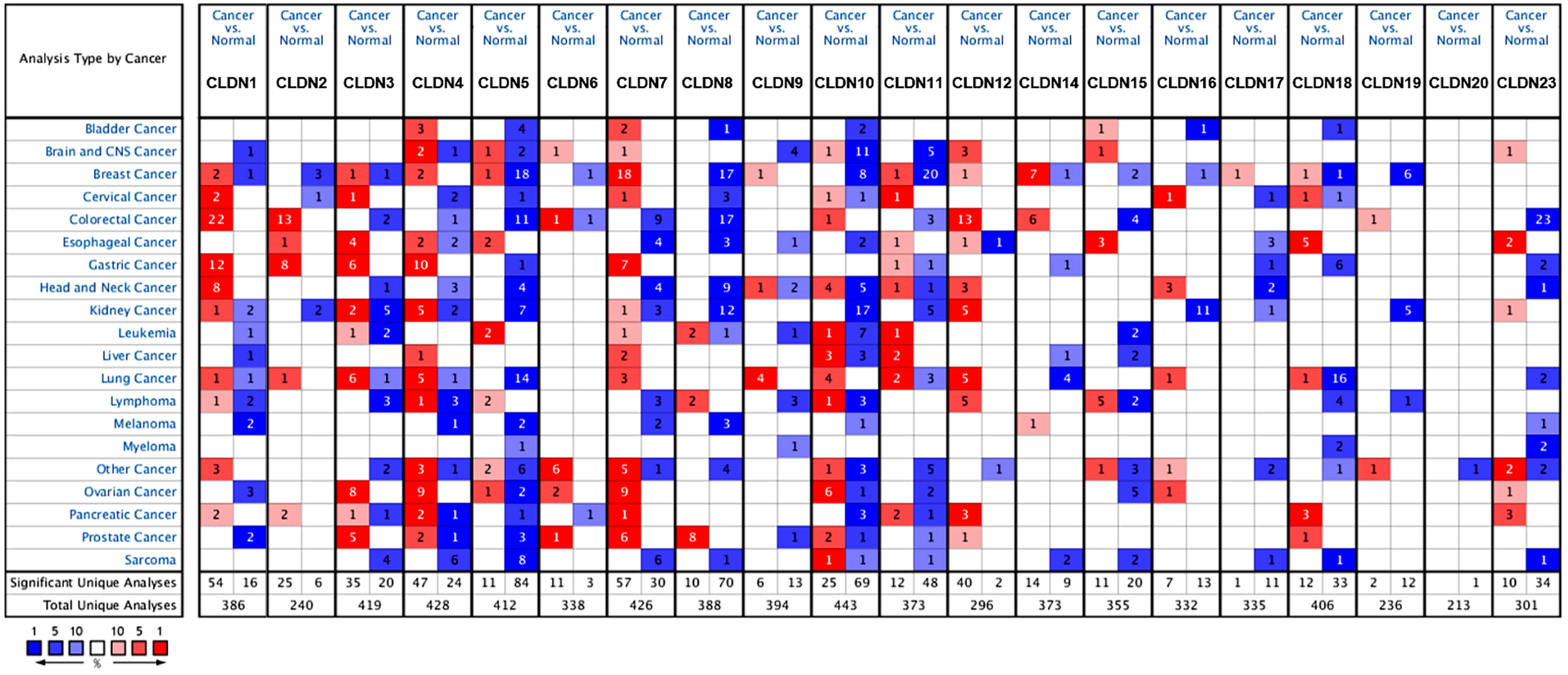


**Supplementary** **Figure S5**. Transcriptional expression of claudins (*CLDNs*) in 20 different types of cancer (Oncomine). Red means of high expression and blue means of low expression. The data was compared with students’ t-test. *P*<0.01, fold change>1.5, gene rank: 10%. It is regrettable that Oncomine database was taken offline on 17 January 2022. Fortunately, the data of CLDNs transcriptional expressions were obtained on 15 February 2021.

**Supplementary Table S1**. The top 10 countries/regions with the strongest productive bursts.

| **Countriy/Region** | **Year** | **Strength** | **Begin** | **End** | **2005 - 2022** |
| --- | --- | --- | --- | --- | --- |
| United States | 2005 | 13.30 | 2005 | 2007 | ▃▃▃▂▂▂▂▂▂▂▂▂▂▂▂▂▂▂ |
| Hungary | 2005 | 12.26 | 2005 | 2011 | ▃▃▃▃▃▃▃▂▂▂▂▂▂▂▂▂▂▂ |
| Norway | 2005 | 3.40 | 2005 | 2013 | ▃▃▃▃▃▃▃▃▃▂▂▂▂▂▂▂▂▂ |
| Finland | 2005 | 6.33 | 2006 | 2014 | ▂▃▃▃▃▃▃▃▃▃▂▂▂▂▂▂▂▂ |
| Japan | 2005 | 4.24 | 2008 | 2008 | ▂▂▂▃▂▂▂▂▂▂▂▂▂▂▂▂▂▂ |
| Spain | 2005 | 3.04 | 2012 | 2016 | ▂▂▂▂▂▂▂▃▃▃▃▃▂▂▂▂▂▂ |
| Egypt | 2013 | 3.40 | 2014 | 2017 | ▂▂▂▂▂▂▂▂▂▃▃▃▃▂▂▂▂▂ |
| Iran | 2013 | 3.14 | 2019 | 2022 | ▂▂▂▂▂▂▂▂▂▂▂▂▂▂▃▃▃▃ |
| India | 2012 | 5.72 | 2020 | 2022 | ▂▂▂▂▂▂▂▂▂▂▂▂▂▂▂▃▃▃ |
| Russia | 2016 | 3.24 | 2021 | 2022 | ▂▂▂▂▂▂▂▂▂▂▂▂▂▂▂▂▃▃ |

**Supplementary Table S2**. The top 10 co-cited authors with the strongest citation bursts.

| **Co-cited Author** | **Year** | **Strength** | **Begin** | **End** | **2005 - 2022** |
| --- | --- | --- | --- | --- | --- |
| Hough CD | 2005 | 19.85 | 2005 | 2011 | ▃▃▃▃▃▃▃▂▂▂▂▂▂▂▂▂▂▂ |
| Long HY | 2005 | 18.08 | 2005 | 2011 | ▃▃▃▃▃▃▃▂▂▂▂▂▂▂▂▂▂▂ |
| Mitic LL | 2005 | 17.82 | 2005 | 2009 | ▃▃▃▃▃▂▂▂▂▂▂▂▂▂▂▂▂▂ |
| Kwon MJ | 2013 | 23.07 | 2015 | 2022 | ▂▂▂▂▂▂▂▂▂▂▃▃▃▃▃▃▃▃ |
| Tabaries S | 2017 | 32.15 | 2017 | 2022 | ▂▂▂▂▂▂▂▂▂▂▂▂▃▃▃▃▃▃ |
| Gunzel D | 2017 | 27.15 | 2017 | 2022 | ▂▂▂▂▂▂▂▂▂▂▂▂▃▃▃▃▃▃ |
| Singh AB | 2011 | 19.36 | 2017 | 2022 | ▂▂▂▂▂▂▂▂▂▂▂▂▃▃▃▃▃▃ |
| Siegel RL | 2017 | 42.76 | 2018 | 2022 | ▂▂▂▂▂▂▂▂▂▂▂▂▂▃▃▃▃▃ |
| Bhat AA | 2018 | 24.52 | 2018 | 2022 | ▂▂▂▂▂▂▂▂▂▂▂▂▂▃▃▃▃▃ |
| Sahin U | 2014 | 26.22 | 2019 | 2022 | ▂▂▂▂▂▂▂▂▂▂▂▂▂▂▃▃▃▃ |

**Supplementary Table S3**. The top 10 productive source journals.

| **Rank** | **Source Journal** | **Publications** | **Citations** | **Citations/****Publication** | **JIF2021** | **Quartile** |
| --- | --- | --- | --- | --- | --- | --- |
| 1 | PLoS One | 43 | 1713 | 39.84 | 3.752 | Q2 |
| 2 | International Journal of Molecular Sciences | 42 | 640 | 15.24 | 6.208 | Q1 |
| 3 | Oncology Reports | 37 | 724 | 19.57 | 4.136 | Q3 |
| 4 | Human Pathology | 28 | 901 | 32.18 | 3.526 | Q2 |
| 5 | American Journal of Surgical Pathology | 27 | 1241 | 45.96 | 6.298 | Q1 |
| 6 | Oncology Letters | 27 | 335 | 12.41 | 3.111 | Q3 |
| 7 | Oncotarget* | 27 | 716 | 26.52 | - | - |
| 8 | Virchows Archiv | 25 | 696 | 27.84 | 4.548 | Q2 |
| 9 | Anticancer Research | 24 | 531 | 22.13 | 2.435 | Q4 |
| 10 | BMC Cancer | 22 | 1049 | 47.68 | 4.638 | Q2 |

* *Oncotarget* was not included in SCI since 2017.

**Supplementary Table S4**. The top 10 co-cited references.

| **Rank** | **Co-cited References Title** | **Journal** | **Citations** | **JIF2021** | **Quartile** |
| --- | --- | --- | --- | --- | --- |
| 1 | Claudin proteins in human cancer: promising new targets for diagnosis and therapy [1] | Cancer Research | 93 | 13.312 | Q1 |
| 2 | The claudin gene family: expression in normal and neoplastic tissues [2] | BMC Cancer | 85 | 4.638 | Q2 |
| 3 | Claudin-3 and claudin-4 expression in ovarian epithelial cells enhances invasion and is associated with increased matrix metalloproteinase-2 activity [3] | Cancer Research | 84 | 13.312 | Q1 |
| 4 | Loss of the tight junction protein claudin-7 correlates with histological grade in both ductal carcinoma in situ and invasive ductal carcinoma of the breast [4] | Oncogene | 83 | 8.756 | Q1 |
| 5 | Claudin-1 regulates cellular transformation and metastatic behavior in colon cancer [5] | Journal of Clinical Investigation | 71 | 19.477 | Q1 |
| 6 | Tight junction proteins claudin-3 and claudin-4 are frequently overexpressed in ovarian cancer but not in ovarian cystadenomas [6] | Clinical Cancer Research | 70 | 9.052 | Q1 |
| 7 | The role of claudins in cancer metastasis [7] | Oncogene | 61 | 8.756 | Q1 |
| 8 | Claudin-4 expression decreases invasiveness and metastatic potential of pancreatic cancer [8] | Cancer Research | 59 | 13.312 | Q1 |
| 9 | Cancer statistics, 2018 [9] | CA-A Cancer Journal for Clinicians | 56 | 286.130 | Q1 |
| 10 | Claudin-1, -3 and -4 proteins and mRNA expression in benign and malignant breast lesions: a research study [10] | Breast Cancer Research | 56 | 8.408 | Q1 |

**Supplementary Table S5**. The most influential potential co-cited reference of each cluster.

| **Rank** | **∑*** | **Co-cited References Title** | **Cluster** | **Citation** | **Burst** | **Centrality** |
| --- | --- | --- | --- | --- | --- | --- |
| 1 | 8.04 | Predicted expansion of the claudin multigene family [11] | #1 ‘triple‒negative breast cancer’ | 46 | 21.81 | 0.1 |
| 2 | 3.46 | Claudin-7 expression induces mesenchymal to epithelial transformation (MET) to inhibit colon tumorigenesis [12] | #2 ‘CLDN7’ | 20 | 8.55 | 0.16 |
| 3 | 3.14 | Structure and function of claudins [13] | #3 ‘cervical carcinoma’ | 34 | 15.87 | 0.07 |
| 4 | 2.99 | Anti-claudin 18.2 antibody as new targeted therapy for advanced gastric cancer [14] | #4 ‘gastric cancer’ | 33 | 15.71 | 0.07 |
| 5 | 2.21 | Decreased expression of claudin-1 correlates with recurrence status in breast cancer [15] | #0 ‘migration’ | 27 | 10.56 | 0.08 |
| 6 | 2.06 | Phenotypic and molecular characterization of the claudin-low intrinsic subtype of breast cancer [16] | #7 ‘breast cancer’ | 45 | 20.53 | 0.04 |
| 7 | 1.88 | The role of claudins in cancer metastasis [7] | #5 ‘CLDN6’ | 61 | 23.33 | 0.03 |
| 8 | 1.18 | Claudin-based tight junctions are crucial for the mammalian epidermal barrier: a lesson from claudin-1–deficient mice [17] | #6 ‘serial analysis of gene expression’ | 17 | 8.63 | 0.02 |
| 9 | 1.16 | The long noncoding RNA TUG1 regulates blood-tumor barrier permeability by targeting miR-144 [18] | #8 ‘blood‒brain barrier’ | 7 | 4.11 | 0.04 |

* ∑, a compound metric computed as (centrality +1)^burstness^ indicating influential potential.

References

1. Morin, P.J. Claudin proteins in human cancer: promising new targets for diagnosis and therapy. *Cancer Res* **2005**, *65*, 9603-9606, doi:10.1158/0008-5472.Can-05-2782.

2. Hewitt, K.J.; Agarwal, R.; Morin, P.J. The claudin gene family: expression in normal and neoplastic tissues. *BMC Cancer* **2006**, *6*, 186, doi:10.1186/1471-2407-6-186.

3. Agarwal, R.; D'Souza, T.; Morin, P.J. Claudin-3 and claudin-4 expression in ovarian epithelial cells enhances invasion and is associated with increased matrix metalloproteinase-2 activity. *Cancer Res* **2005**, *65*, 7378-7385, doi:10.1158/0008-5472.Can-05-1036.

4. Kominsky, S.L.; Argani, P.; Korz, D.; Evron, E.; Raman, V.; Garrett, E.; Rein, A.; Sauter, G.; Kallioniemi, O.-P.; Sukumar, S. Loss of the tight junction protein claudin-7 correlates with histological grade in both ductal carcinoma in situ and invasive ductal carcinoma of the breast. *Oncogene* **2003**, *22*, 2021-2033, doi:10.1038/sj.onc.1206199.

5. Dhawan, P.; Singh, A.B.; Deane, N.G.; No, Y.; Shiou, S.R.; Schmidt, C.; Neff, J.; Washington, M.K.; Beauchamp, R.D. Claudin-1 regulates cellular transformation and metastatic behavior in colon cancer. *J Clin Invest* **2005**, *115*, 1765-1776, doi:10.1172/jci24543.

6. Rangel, L.B.; Agarwal, R.; D'Souza, T.; Pizer, E.S.; Alò, P.L.; Lancaster, W.D.; Gregoire, L.; Schwartz, D.R.; Cho, K.R.; Morin, P.J. Tight junction proteins claudin-3 and claudin-4 are frequently overexpressed in ovarian cancer but not in ovarian cystadenomas. *Clin Cancer Res* **2003**, *9*, 2567-2575.

7. Tabariès, S.; Siegel, P.M. The role of claudins in cancer metastasis. *Oncogene* **2017**, *36*, 1176-1190, doi:10.1038/onc.2016.289.

8. Michl, P.; Barth, C.; Buchholz, M.; Lerch, M.M.; Rolke, M.; Holzmann, K.H.; Menke, A.; Fensterer, H.; Giehl, K.; Löhr, M.; et al. Claudin-4 expression decreases invasiveness and metastatic potential of pancreatic cancer. *Cancer Res* **2003**, *63*, 6265-6271.

9. Siegel, R.L.; Miller, K.D.; Jemal, A. Cancer statistics, 2018. *CA Cancer J Clin* **2018**, *68*, 7-30, doi:10.3322/caac.21442.

10. Tokés, A.M.; Kulka, J.; Paku, S.; Szik, A.; Páska, C.; Novák, P.K.; Szilák, L.; Kiss, A.; Bögi, K.; Schaff, Z. Claudin-1, -3 and -4 proteins and mRNA expression in benign and malignant breast lesions: a research study. *Breast Cancer Res* **2005**, *7*, R296-305, doi:10.1186/bcr983.

11. Mineta, K.; Yamamoto, Y.; Yamazaki, Y.; Tanaka, H.; Tada, Y.; Saito, K.; Tamura, A.; Igarashi, M.; Endo, T.; Takeuchi, K.; et al. Predicted expansion of the claudin multigene family. *FEBS Letters* **2011**, *585*, 606-612, doi:<https://doi.org/10.1016/j.febslet.2011.01.028>.

12. Bhat, A.A.; Pope, J.L.; Smith, J.J.; Ahmad, R.; Chen, X.; Washington, M.K.; Beauchamp, R.D.; Singh, A.B.; Dhawan, P. Claudin-7 expression induces mesenchymal to epithelial transformation (MET) to inhibit colon tumorigenesis. *Oncogene* **2015**, *34*, 4570-4580, doi:10.1038/onc.2014.385.

13. Krause, G.; Winkler, L.; Mueller, S.L.; Haseloff, R.F.; Piontek, J.; Blasig, I.E. Structure and function of claudins. *Biochimica et Biophysica Acta (BBA) - Biomembranes* **2008**, *1778*, 631-645, doi:<https://doi.org/10.1016/j.bbamem.2007.10.018>.

14. Singh, P.; Toom, S.; Huang, Y. Anti-claudin 18.2 antibody as new targeted therapy for advanced gastric cancer. *Journal of Hematology & Oncology* **2017**, *10*, 105, doi:10.1186/s13045-017-0473-4.

15. Morohashi, S.; Kusumi, T.; Sato, F.; Odagiri, H.; Chiba, H.; Yoshihara, S.; Hakamada, K.; Sasaki, M.; Kijima, H. Decreased expression of claudin-1 correlates with recurrence status in breast cancer. *Int J Mol Med* **2007**, *20*, 139-143.

16. Prat, A.; Parker, J.S.; Karginova, O.; Fan, C.; Livasy, C.; Herschkowitz, J.I.; He, X.; Perou, C.M. Phenotypic and molecular characterization of the claudin-low intrinsic subtype of breast cancer. *Breast Cancer Research* **2010**, *12*, R68, doi:10.1186/bcr2635.

17. Furuse, M.; Hata , M.; Furuse , K.; Yoshida , Y.; Haratake , A.; Sugitani , Y.; Noda , T.; Kubo , A.; Tsukita , S. Claudin-based tight junctions are crucial for the mammalian epidermal barrier : a lesson from claudin-1–deficient mice. *Journal of Cell Biology* **2002**, *156*, 1099-1111, doi:10.1083/jcb.200110122.

18. Cai, H.; Xue, Y.; Wang, P.; Wang, Z.; Li, Z.; Hu, Y.; Li, Z.; Shang, X.; Liu, Y. The long noncoding RNA TUG1 regulates blood-tumor barrier permeability by targeting miR-144. *Oncotarget* **2015**, *6*, 19759-19779, doi:10.18632/oncotarget.4331.
